# Serous cystadenocarcinoma of the mesentery in a man: case report and review of literature

**DOI:** 10.1093/gastro/gou019

**Published:** 2014-04-07

**Authors:** Toru Obuchi, Osamu Shimooki, Akira Sasaki, Tadashi Abe, Go Wakabayashi

**Affiliations:** ^1^ Department of Surgery, Iwate Medical University School of Medicine, Iwate, Japan; ^2^ Department of Surgery, Iwate Prefectural Kuji Hospital, Iwate, Japan

**Keywords:** Gastrointestinal surgery, immunohistochemical, mesenteric cyst, cystadenocarcinoma

## Abstract

In February 2007, a 41-year-old Japanese male was admitted to our hospital with increasing upper abdominal pain. A contrast-enhanced computed tomography (CT) scan of the abdomen demonstrated a well-demarcated, hypodense cystic mass with a thickened wall in the mesocolon. The laboratory results were within normal limits, except for increased carcinoembryonic antigen, carbohydrate antigen 19-9, DUPAN-2 and SPAN-1. The patient was diagnosed as having a mesenteric malignant cyst, and during a laparotomy, a right hemicolectomy with mesenteric cystectomy was performed without rupture in March 2007. In the microscopic findings, there was a well-differentiated adenocarcinoma in the inner surface of the cyst and in the fibrous connective tissue of the hypertrophic cystic wall. The tumor cells were immunohistochemically reactive to cytokeratin (CK) 7, CK18 and CK20. No remnant of the malignancy was detected in the resected margin of the colon, cyst, liver or peritoneum nor was an uptake detected in an 18[F]-fluorodeoxyglucose positron emission tomography/CT examination of other organs. Finally, the malignancy was concluded to be a serous cystadenocarcinoma of the mesentery. Nineteen months after the operation, the patient died from peritonitis carcinomatosa due to a small intestine rupture. This report suggests mesenteric cystadenocarcinomas originating in the ovary, oviduct and intestinal mucosa, but these were ruled out in our patient. In this report, we discuss a case of the malignant transformation of a cyst into adenocarcinoma, which to our knowledge has never been previously reported in a male patient.

## INTRODUCTION

Mesenteric cystadenocarcinomas originating in the ovary, oviduct, and intestinal mucosa have been reported [1–2], but these were ruled out in our male patient. Only four female patients with this disease have been reported [3–6], and this is the first in which a bilateral appendage-related origin could be ruled out and added the immunohistochemical result (i.e. cytokeratin), which to our knowledge has never been previously reported in a male case.

## CASE REPORT

**Figure 1.**
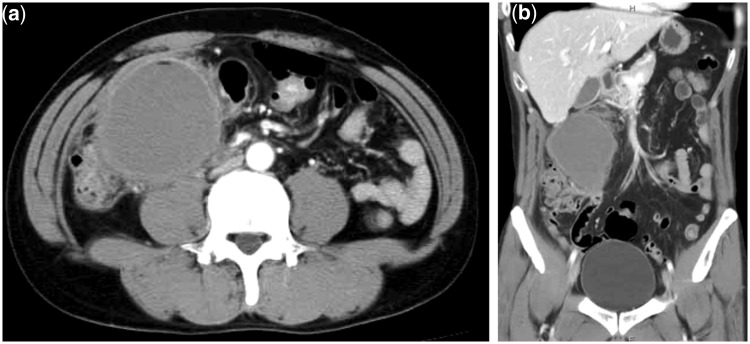
A contrast medium-enhanced computed tomography showing a large, unilocular cystic mass in the right retroperitoneal space: (**a**) axial view; (**b**) coronal view.

In February 2007, a 41-year-old Japanese male presented with upper abdominal pain. A computed tomography (CT) showed a large, round unilocular boundary cyst in the mesocolon (Figure 1). The patient was diagnosed as having a mesenteric cyst, and a right hemicolectomy with a mesenteric cystectomy was performed in March 2007. During the laparotomy, the mass was found to be fixed within the ascending colonic mesentery. A part of the peritoneum of the abdominal wall and the edge of the liver (S5) were resected due to adhesion with the cyst (Figure 1). We want to give special emphasis to the fact that the pancreas and biliary tract were free from the cystadenocarcinoma of the mesentery, and the post-operative course was uneventful.

**Figure 2.**
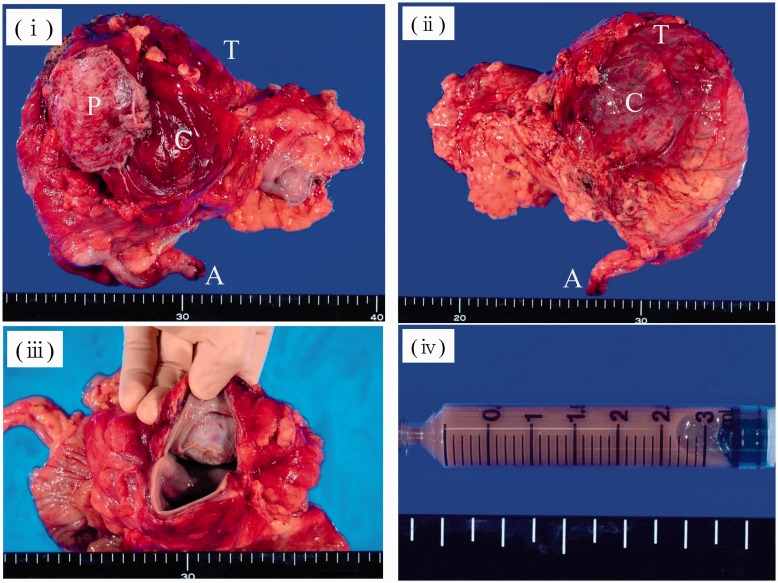
Gross findings of a serous cystadenocarcinoma of the mesentery: (**i**) ventral side; (**ii**) dorsal side; (**iii**) A cross-section; (**iv**) the fluid in the cyst. A = appendix; C = cyst; L = liver (S5); P = peritoneum; T = transverse colon.

**Figure 3.**
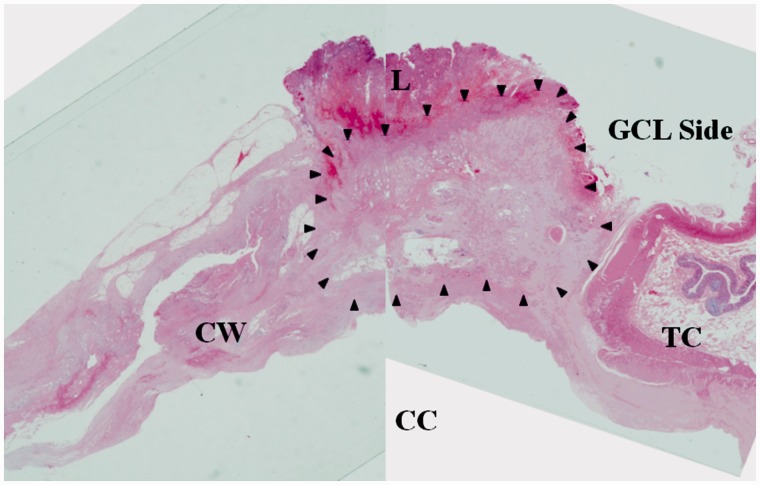
Low-magnification photo of the adenocarcinoma in the cyst (arrowhead). CC = cystic cavity; CW = cystic wall; GCL = gastrocolic ligament; L = liver; TC = transverse colon.

**Figure 4.**
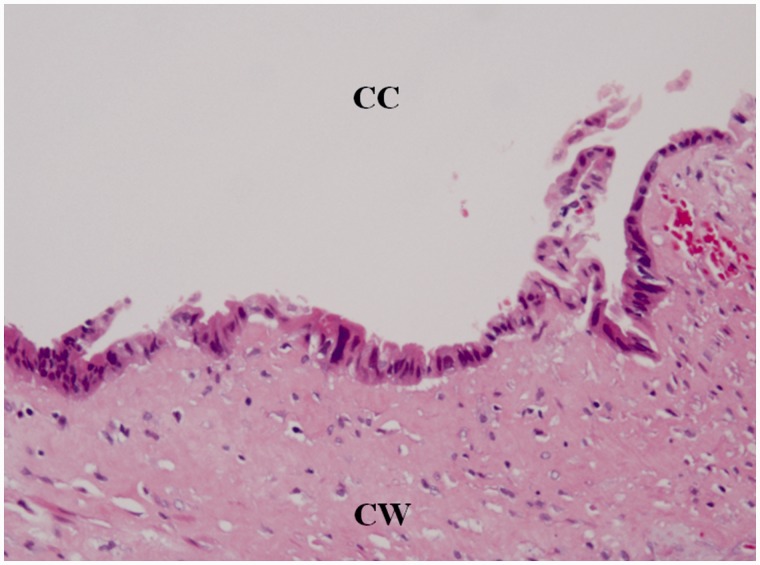
High-magnification photo of the adenocarcinoma existing in the inner surface of the cyst. CC = cystic cavity; CW = cystic wall.

**Figure 5.**
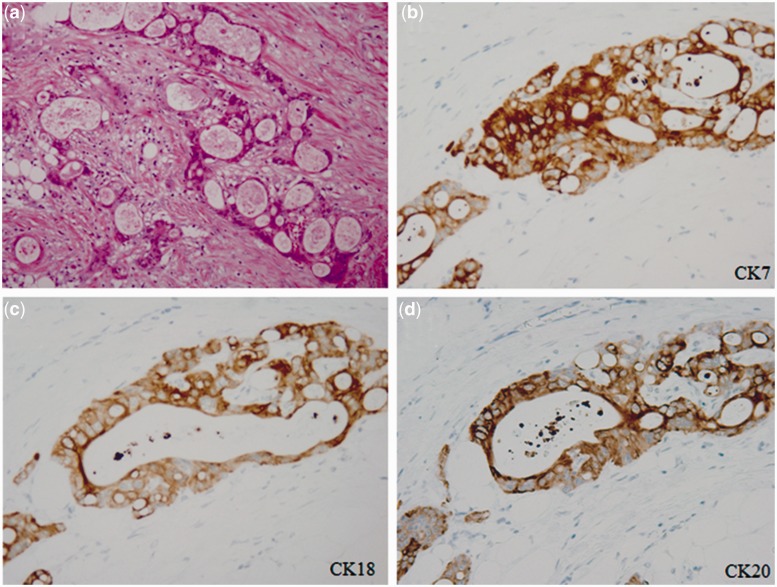
Histological findings of the cystic wall (hematoxylin and eosin stain, higher magnification); fibrous thick wall having adenocarcinoma without epithelium (**a**) the tumor cells were immunohistochemically reactive for CK7 (**b**), CK18 (**c**), and CK20 (**d**).

The resected specimen measured 7.5 × 8.6 × 9.5 cm in size; the wall of the cyst was 3 mm thick (Figure 1) and the cyst contained a serous, cloudy ‘café-au-lait'-like fluid (Figure 2). A histopathological examination revealed that the main location of the cancer was close to the partial hepatectomy, without direct invasion of the liver and the main tumor was free from the ascending/transverse colon (Figure 3). There was a well-differentiated adenocarcinoma in the inner surface of the cyst and in the fibrous connective tissue of the hypertrophic cystic wall. No remnant of malignancy was detected in the resected margin of the colon, cyst, liver or peritoneum. There was a well-differentiated adenocarcinoma in the inner surface of the cyst and in the fibrous connective tissue of the hypertrophic cystic wall. None of the benign epithelium was seen in the inner surface of the cyst: it was speculated that it had been left out, and the epithelium was only seen near the partial hepatectomy in the cystic wall (Figure 4). The tumor cells were immunohistochemically reactive for CK7, CK18, and CK20 (Figure 5).

Although a histological diagnosis of this case seems to be difficult, the primary site of this adenocarcinoma was assumed to be the peritoneum, because the primary lesions were found in no other organs during the operation. No remnant of malignancy was detected in the resected margin of the liver. Finally, we diagnosed cystadenocarcinoma of the mesocolon. Post-operative positron emission tomography(PET)-CT scan showed no detectable tumor cells in other organs. At five months after operation, the serum concentration of CEA and carbohydrate antigen 19-9(CA19-9) had decreased to within normal limits. At 19 months after operation, the patient was dead from acute panperitonitis due to small intestine rupture. In autopsy, recurrence of adenocarcinoma was found in the retroperitoneum, involving the hepatic hilum with perineural invasion and the pancreas head. Metastasis to fat tissue around the ureter had caused obstructive hydronephrosis. Adenocarcinoma of the peritoneal cavity had recurred extensively, resulting in lesions occupying the peritoneal cavity, which finally caused the man’s death. Although histological diagnosis of this case seems to be difficult, the primary site of this adenocarcinoma is assumed be the peritoneum, because primary lesions were found in no other organs at operation or autopsy.

## DISCUSSION

There is no consensus on the exact origin of cystadenocarcinoma of the mesentery, but it has been attributed to accidental implantations, malformation, the ovary, and the intestine [5]. Only 19 cases of primary cystadenocarcinoma of the mesentery have been reported in the world. Mesenteric cysts occur in patients of all ages, but they are considerably more common in women, and mesenteric mucinous cystic neoplasms (MCN) are simply regarded as extra-ovarian MCNs [7]. We investigated four cases of serous cystadenocarcinoma of the mesentery, excluding those of gynecological origin (Table 1; [3–6]). The origins could not be determined in these cases, but an ovarian origin could not be ruled out in two of the four cases. This male case is very interesting because an origin related to the bilateral appendages, such as reported cases associated with ovarian tumors, Mullerian cysts [1], and endosalpingiosis [2], can be ruled out. A close comparison with previous reports is difficult because no immunostaining was performed in any of the reported cases. In the histological diagnosis of this case, teratoma, lymphangioma and ovarian tumors were denied by the microscopic findings. The surgical findings did not detect an origin in the liver, gall bladder or pancreas, and no other adhesions could be detected in the abdominal cavity, with the exception of a mesenteric cyst. This cyst adhered to the peritoneum and the liver, which suggests that the patient was unlikely to have met with serious external injuries; however, local external injuries might have occurred.

**Table 1.** Resected cases of cystadenocarcinoma of the mesentery

Author	Year	Age/Gender	Chief complaints	Treatment	Histopathology	Suspected origin	Prognosis
Peterson *et al.* [5]	1933	36/ Female	Abdominal tumor	Cystectomy	Papillary adenocarcinoma	Ovarian or intestinal tissue	Unknown
Tykka *et al.* [4]	1975	23/ Female	Bloody stool	Left hemicolectomy	Papillary adenocarcinoma	Ovarian	Unknown
Harakawa *et al.* [6]	1986	34/ Female	Abdominal tumor	Right hemicolectomy	Papillary adenocarcinoma	Intestinal tissue	Alive/ POM 9
Bury *et al.* [3]	1994	36/ Female	Unknown	Cystectomy	Adenocarcinoma	TBM	Dead/ POM 16
Our case	2014	41/ Male	Abdominal pain	Right hemicolectomy	Well-differentiated adenocarcinoma	TBM	Dead/ POM 19

POM = post operative month; TBM = transformation of benign mesenteric cyst.

Our views on the reason why the pancreatico-biliary epithelia created a mesenteric cyst are as follows. Firstly, it seems possible that an implantation of a pancreatico-biliary fragment from an external injury could have metastasized in the mesentery and increased in volume as a cystic formation with a cancerous change of the normal cells. The cyst was filled with cloudy ‘café-au-lait' fluid; chylous cysts are usually associated with small bowel mesentery and serous cysts with the mesocolon, and hemorrhagic cysts are caused by trauma [8]. Therefore, the cyst may have been formed as the result of an external force that the patient did not remember. Secondly, we considered the possibility that the cystadenocarcinoma was identical to a pancreatico-biliary tumor *de novo*, because of the similarities in the pathological immunochemical findings. These findings permit a good guess at the origin, but not a perfect one; none has been reported to date and, notably, this case is the first to be examined with an immunohistochemical approach. The cyst could have metastasized in the mesentery and increased in volume as a cystic formation with an epithelium like a positive pancreatico-biliary marker with a cancerous change of the normal cells. Finally, the benign mesenteric cyst might have transformed into cystadenocarcinoma of the mesocolon after the first or second scenarios described above.

The recurrence pattern of previously reported cases was unclear, but peritonitis carcinomatosa associated with peritoneal dissemination occurred in our patient. Also, the surgical margin was negative in our patient, but there was a thin margin in the surgical cut-line of the gastrocolic ligament (Figure 3). Therefore, the reason for the recurrence after radical surgery was the skip-lesion of the cancer, because it recurred adjacent to the original location of the cancer, suggesting that the pre-renal fascia and the gastrocolic ligament should also be resected with sufficient margin, in addition to resection of the cyst including the mesentery. We would like to examine this further with case reports similar to our abstruse case.

**Conflict of interest:** none declared.
